# Cerebral Blood Flow and its Connectivity Deficits in Patients With Lung Cancer After Chemotherapy

**DOI:** 10.3389/fmolb.2022.761272

**Published:** 2022-03-23

**Authors:** Yujie Zhang, Song’an Shang, Lanyue Hu, Jia You, Wei Gu, Vijaya Prakash Muthaiah, Yu-Chen Chen, Xindao Yin

**Affiliations:** ^1^ Department of Radiology, Nanjing First Hospital, Nanjing Medical University, Nanjing, China; ^2^ Department of Respiratory Medicine, Nanjing First Hospital, Nanjing Medical University, Nanjing, China; ^3^ Department of Rehabilitation Science, School of Public Health and Health Professions, University at Buffalo, Buffalo, NY, United States

**Keywords:** chemotherapy, cognitive impairment, lung cancer, cerebral blood flow, arterial Spin labeling

## Abstract

**Purpose:** This study was performed to investigate the regional cerebral blood flow (CBF) and CBF connectivity in the chemotherapy-induced cognitive impairment of patients with lung cancer by using arterial spin labeling.

**Methods:** Pseudocontinuous arterial spin labeling perfusion magnetic resonance imaging and neuropsychological tests were performed for 21 patients with non-small cell lung cancer who had received chemotherapy CT (+) and 25 non-small cell lung cancer patients who need chemotherapy but did not yet received CT (-). The CT (+) group previously received platinum-based therapy for 3 months to 6 months (the time from their first chemotherapy to the MRI scan). Group comparisons were performed in the regional normalized CBF and CBF connectivity, and the relationship between the regional normalized CBF and cognitive impairment were detected.

**Results:** The CT (+) group exhibited higher CBF in the left insula, right caudate, right superior occipital gyrus, left superior temporal gyrus (STG), and right middle frontal gyrus (MFG). MoCA scores as well as the memory scores were negatively correlated with the increased CBF in the right MFG (*r* = −0.492, *p* = 0.023; *r* = −0.497, *p* = 0.022). Alterations in the CBF connectivity were detected only in the CT (+) group between the following: right MFG and the right precentral gyrus; the right caudate and the right lingual gyrus; right caudate and right precuneus; left STG and the bilateral MFG; and the left STG and the right middle cingulum.

**Conclusion:** These findings indicated that chemotherapy is associated with abnormalities in the CBF and connectivity alterations, which may contribute to the cognitive impairment in patients with lung cancer.

## Introduction

Chemotherapy-induced cognitive impairment (CRCI) occurs in patients with cancer in the non-central nervous system who are exposed to chemotherapy or other anticancer treatments (Mounier et al., 2020). Previous studies have demonstrated that patients with lung cancer during or post-chemotherapy suffer with pronounced cognitive impairment, which can reduce the quality of life (Simó et al., 2018; Mentzelopoulos et al., 2021). These results remain controversial, and the neuroimaging basis of these findings needs more exploration. Lung cancer is the most common malignant tumor, accounting for 11.6% of the global total cancer (Siegel et al., 2018). Platinum drugs are the first-line chemotherapy drug for non-small cell lung cancer with effective result. In recent years, the survival rates of patients with lung cancer have improved as a result of appropriate treatment and extensive use of platinum drugs. According to the latest statistics of the American Cancer Society, among cancer types, lung cancer has the most significant reduction in death rates, with a 51% decrease in males and a 26% decrease in females (Siegel et al., 2020). Due to the prolongation of survival and the effect of drugs, CRCI of survivors of lung cancer has been a serious issue in recent years.

Previous studies on CRCI in patients with lung cancer have mainly concentrated on the changes in the structure and function of the brain. Structural studies have revealed reduced gray matter and white matter volumes in the cortical and subcortical brain regions following chemotherapy, including several default mode network (DMN) regions (Pomykala et al., 2013; Simó et al., 2016; ırak et al., 2020). Functional studies have demonstrated the reduced resting-state FC pattern within the DMN and the cognitive disorder are associated with cancer and chemotherapy in patients with lung cancer (Simó et al., 2018; Zhang Y. et al., 2020). Hu et al. (2020) explored links between changes in the intrinsic static and dynamic functional connectivity in the executive control network (ECN) patterns and chemotherapy in patients with lung cancer. For such controversial results, more advanced research methods should be used to solve these problems.

Few studies based on positron emission tomography and single-photon emission computed tomography (SPECT) have shown abnormal cerebral blood flow (CBF) and cerebral glucose metabolism in multiple brain regions of breast cancer survivors treated with chemotherapy (Silverman et al., 2007; Ponto et al., 2015). PET and SPECT demand the use of invasive radioactive tracers, which makes the repeated detection difficult. Arterial spin labeling (Aslan and Lu, 2010) provides the noninvasive quantification of CBF, and this process labels the water in the artery as an endogenous tracer with reverse pulses (Alsop et al., 2015), which can eliminate expensive and harmful radioactive substances and quantitatively measure the CBF. ASL has been employed to explore the resting-state perfusion alterations after chemotherapy in patients with breast cancer. Results demonstrate that regional CBF in the right precentral gyrus was elevated, while in the bilateral frontal and parietal lobes, the regional CBF showed a decrease (Nudelman et al., 2014; Nudelman et al., 2016). Chemotherapy-related changes in the CBF may be caused by mechanisms of tissue compensation after the chemotherapy-induced damage of cells, blood vessels, and tissues (Feng et al., 2019). We also infer that these changes will occur in patients with lung cancer after chemotherapy, which needs further investigation.

CBF of different brain regions is not independent, which can reflect the changes of neuronal activity. Synchronous changes of CBF connectivity may occur in areas of the same functional network (Havsteen et al., 2018; Jaganmohan et al., 2021). The changes in CBF-connectivity were explored in a variety of neurological diseases, such as Alzheimer’s disease (Zheng et al., 2019) and Parkinson’s disease (Shang et al., 2021). CBF connectivity changes in patients with lung cancer after chemotherapy is still not clear and needs further exploration. Therefore, our research was designed to explore aberrant CBF-associated changes caused by platinum-based therapy in non-small cell lung cancer using ASL. We hypothesized that platinum-based therapy influences CBF pattern and CBF connectivity in non-small cell lung cancer patients; ASL-based perfusion may serve as a noninvasive *in vivo* biomarker related to CRCI in non-small cell lung cancer.

## Materials and Methods

### Participants

A total of 21 post-chemotherapy CT (+) and 25 pre-chemotherapy CT (–) patients with stage II to III lung cancer were selected from the Department of Respiratory Medicine, Nanjing First Hospital from September 2018 to August 2020. All participants enrolled in the study were 55–70 years of age, right-handed, and had at least 6 years of education. The groups were matched for age, gender, and education. All the participants underwent surgery. Both groups were pathologically diagnosed with non-small cell lung cancer without any other antitumor treatment, including targeted therapy, immunotherapy, and radiation therapy. Both groups accept Performance Status Assessment (PS) (Dhillon et al., 2017) and Quality of Life Assessment (QoL) (Huang et al., 2017). The CT (+) group previously received platinum-based therapy for 3 months to 6 months (the time from their first chemotherapy to the MRI scan, 136.85 ± 27.01 d). Regimen for the CT (+) group included cisplatin + pemetrexed (8 patients), cisplatin + docetaxel (3 patients), cisplatin + gemcitabine (4 patients), cisplatin + etoposide (3 patients), and pemetrexed + paraplatin (3 patients). No participants were excluded from the fMRI analysis due to excessive head motion during the scanning. The study was approved by the Ethics Committee of Nanjing Medical University. All individuals provided informed written consent before the study. Exclusion criteria were: 1) small cell lung cancer, stage I and IV non-small cell lung cancer, 2) brain metastatic tumors, 3) prophylactic cranial irradiation, 4) any neurological and psychiatric diseases (according to clinical assessment), such as stroke, Alzheimer’s disease, Parkinson’s disease, depression, dysthymic disorder, 5) major medical illnesses such as thyroid dysfunction, anemia, severe heart diseases and damaged liver or kidney function, severe visual or hearing loss, 6) MR contraindications, and 7) a poor quality of the imaging data. Clinical neurocognitive status of all participants was quantified using the Montreal Cognitive Assessment (MoCA) (Nasreddine et al., 2005). It is used to evaluate several aspects of cognitive function, including attention, language, abstraction, visuospatial/executive, naming, memory and location. All subjects underwent the same MRI scan and cognitive function assessment.

### MRI Data Acquisition

All participants were scanned on 3.0-T MR system (Ingenia, Philips Medical Systems, Netherlands) with an 8-channel digital head coil receiver. The participants used earplugs and were instructed to lie supine while staying awake and closing their eyes without thinking about anything during scans. ASL uses 2D pseudocontinuous ASL (pCASL) sequences to obtain images, and the parameters are as follows: repetition time (TR) 4000 ms; echo time (TE) 11 ms; flip angle = 90°, label duration 1650 ms; post-labeling delay 2000 ms; field of view 240 mm × 240 mm; slice thickness 4 mm with 10% gap; matrix 64 × 64; 20 axial slices; total scan duration 4 min 08 s. Each subject contained 60 volumes and used 30 label-control image pairs. 3D turbo fast echo T1WI sequence was used to obtain Sagittal 3D T1-weighted images, and the parameters are as follows: repetition time 8.1 ms; echo time 3.7 ms; flip angle 8°; field of view 256 mm × 256 mm; acquisition matrix 256 × 256; slice thickness 1 mm; gap 0 mm; and 172 sagittal slices; total scan duration 5 min 28 s.

### MRI Data Processing and CBF Calculation

The CBF maps were calculated based on pCASL data by using ASL data processing toolbox: ASLtbx (https://cfn.upenn.edu/∼zewan) (Wang, 2012). Statistical parameter mapping software (SPM8, http://www.fil.ion.ucl.ac.uk/spm/software/spm8/) and ASLtbx were used to analyze the ASL imaging data. In order to correct the head movement, we rearranged and adjusted control and labeled ASL images.

The CBF images of CT (-) patients were performed on a nonlinear transformation by SPM8 software and coregistered with the positron emission tomography perfusion template of Montreal Neurological Institute (MNI) space. The MNI standard CBF template is defined as the average coregistered CBF image of CT (-) patients. Then the two groups of CBF images coregistered to the MNI-standard CBF template. Each coregistered CBF was removed from non-brain tissue and spatially smoothed with a Gaussian distribution of 8 mm full width half maximum. Normalization is achieved by dividing the CBF per voxel by the average CBF of the whole brain.

SPM8 software was used to perform a nonlinear transformation of the CBF images of the CT (-) patients, and these images were coregistered with the positron emission tomography-perfusion template in the Montreal Neurological Institute (MNI) space. The MNI-standard CBF template was defined as the average coregistered CBF images of the CT (-) patients. The CBF images of the two groups were then coregistered to the MNI-standard CBF template. We removed each coregistered CBF from the non-brain tissue and smoothed with a Gaussian of 8 mm × 8 mm full width at half maximum. Normalization was performed by dividing the CBF per voxel by the average CBF across the entire brain.

### CBF Connectivity Analyses

**FIGURE 1:**
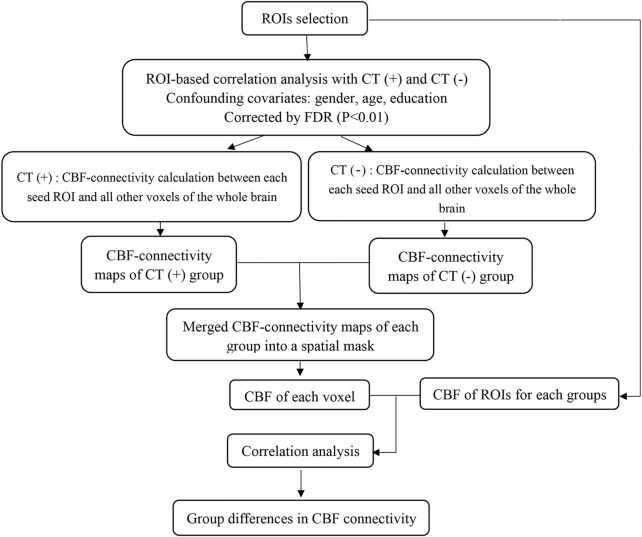
Processing steps of CBF connectivity analyses.

To detect whether abnormalities in the CBF connectivity exist in the brain regions with changes in the CBF in patients with lung cancer post-chemotherapy, we selected the clusters with significant group differences in the CBF as seed regions of interest (ROIs) (Melie-García et al., 2013). Referring to previous studies (Shang et al., 2021), we extracted the CBF values of each ROI within each subject from a separate CBF map. The multiple regression model was used to calculate CBF connections between each ROI seed and all other voxels in the entire brain between individuals for each group. Gender, age, and education were used as confounding covariates. Statistical analyses were used to identify voxels in each group with a positive or negative correlation between the CBF values and CBF values for each seed ROI. Multiple comparisons were corrected using false discovery rate (FDR) correction (*p* < 0.01). The CBF connected graphs for each group were inosculated into a spatial mask, and the CBF of each voxel was then associated with the CBF of the two sets of ROIs. CBF correlation between two groups may have different slopes for any pair of voxels. Different slopes reflect different CBF connectivity. In order to map voxels with significantly different CBF correlations of each seed ROI between the two groups, a specific T comparison was established within the spatial mask of the CBF connectivity map of ROI. Figure 1 shows the whole processing steps.

### Statistical Analysis

Differences in the demographic and clinical data between CT (-) and CT (+) group were analyzed using two-sample t-tests and χ^2^ tests by the SPSS 19.0 software package (*p <* 0.05 was considered significant). Two-sample *t*-test was used for the regional normalized CBF and CBF-connectivity analysis with age, gender, and education level corrected. The significance statistical threshold was set to *p* < 0.01, using FDR correction. For each subject, the regional normalized CBF of each cluster with significant differences between groups was extracted for ROI-based analysis. The correlations between these significant regions (regional CBF values) and neuropsychological performance (MoCA) in two group were investigated using Spearman correlation analysis in SPSS 19.0 software, and the data were corrected for age, sex, and educational level. *p* < 0.05 was defined as statistically significant.

## Results

### Participants and Clinical Data

Table 1 shows patient demographics and clinical data. Differences in the age, sex, education level, PS, QoL, and smoking between groups were not significant (all *p* > 0.05). Compared with the CT (-) group, the CT (+) group showed significantly lower MoCA scores (*p* < 0.001). Among all subcategories of the MoCA, only the memory scores (*p* = 0.002) were significantly lower in the CT (+) group than in the CT (-) group.

**TABLE 1:** Demographic and clinical characteristics of the post-chemotherapy patients and pre-chemotherapy patients.

Characteristic	Post-chemotherapy (*n* = 21)	Pre-chemotherapy (*n* = 25)	*p*-value	ES
Age (years)	65.09 ± 4.96	63.12 ± 6.31	0.108	0.17
Education (years)	7.86 ± 2.01	8.52 ± 2.50	0.334	−0.14
Gender (female/male)	10/11	13/12	0.569	0.66
Smoking	16	17	0.539	0.35
Tumor stage				
II	12	13		
III	9	12		
PS scores	1.14 ± 0.36	1.24 ± 0.44	0.099	−0.12
QoL scores	47.52 ± 4.88	48.52 ± 5.14	0.810	−0.09
MoCA scores	20.29 ± 2.22	23.76 ± 1.81	<0.001^*^	−0.65
Visuospatial/executive	2.67 ± 0.79	3.30 ± 0.91	0.052	−0.34
Naming	1.83 ± 0.68	2.66 ± 0.44	0.062	−0.58
Attention	4.19 ± 1.25	4.60 ± 1.15	0.255	−0.16
Language	2.19 ± 0.51	2.24 ± 0.52	0.748	−0.04
Abstraction	1.84 ± 0.58	2.06 ± 0.45	0.191	−0.21
Memory	2.62 ± 1.29	3.36 ± 1.33	0.002^*^	−0.27
Orientation	5.33 ± 0.91	5.36 ± 0.81	0.917	−0.02

The data are shown as the mean ± SD; MoCA, Montreal Cognitive Assessment; PS, Performance Status Assessment; QoL, Quality of Life Assessment; ES, effect size. ^*^ *p* < 0.05.

### Group Differences in the Regional Normalized CBF

**FIGURE 2:**
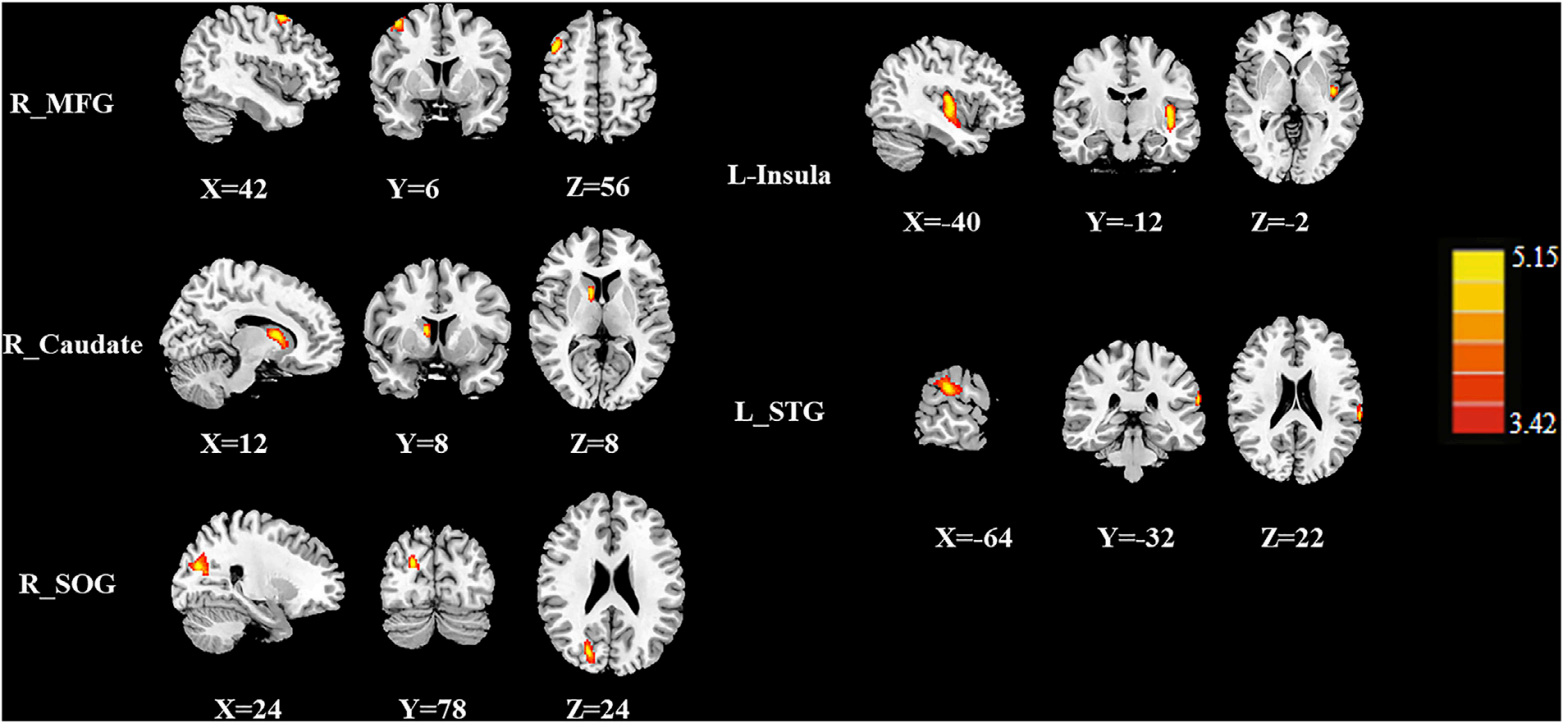
The CBF differences between the post-chemotherapy (CT+) and pre-chemotherapy (CT-) lung cancer patients. Compared with CT (-), the CT (+) group showed higher rCBF in the right middle frontal gyrus (MFG), right caudate, right superior occipital gyrus (SOG), left insula, and left superior temporal gyrus (STG).

**FIGURE 3:**
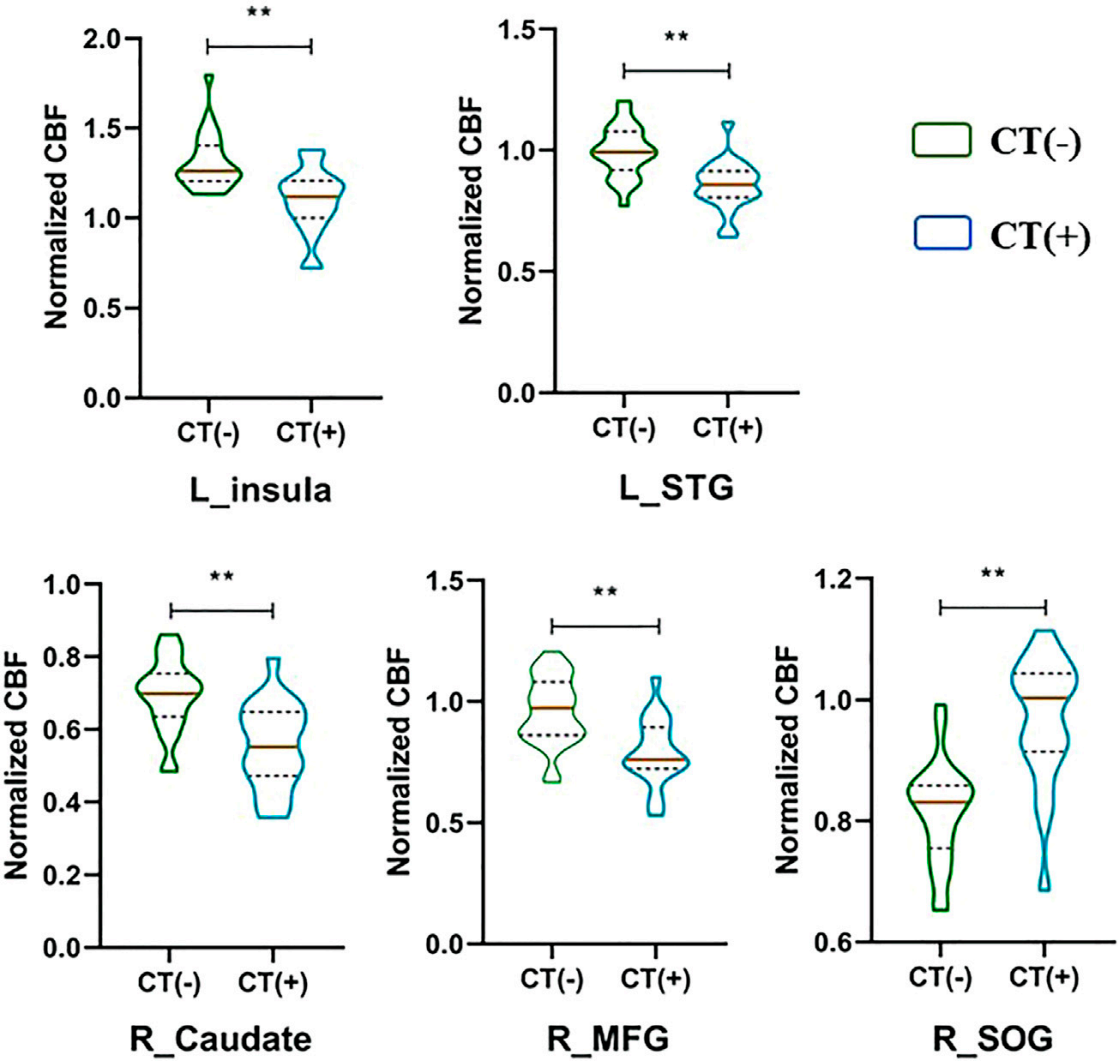
Normalized CBF of brain regions with significant group differences. **p < 0.01.

Figures 2 and 3 and Table 2 show the normalized regional CBF value (rCBF) differences between the two groups. Compared with the CT (-) group, the CT (+) group showed higher CBF in the left insula, right caudate, right superior occipital gyrus (SOG), left superior temporal gyrus (STG), and right middle frontal gyrus (MFG).

**TABLE 2:** Brain regions with significant group differences in normalized CBF.

Brain regions	BA	Normalized CBF		Peak MNI coordinates	Peak T value	Voxels
		CT (-)	CT (+)	x, y, z (mm)		
R_MFG	6	0.859 ± 0.038	0.746 ± 0.017	42, 6, 56	4.032	122
R_Caudate	–	0.610 ± 0.053	0.466 ± 0.086	12, 8, 8	4.910	144
R_SOG	18	0.832 ± 0.029	0.975 ± 0.039	24, -78, 24	5.148	266
L_Insula	21	1.295 ± 0.131	1.007 ± 0.070	-40, -12, -2	4.492	303
L_STG	22	1.028 ± 0.079	0.898 + 0.027	-64, -32, 22	4.760	109

Thresholds were set at a corrected *p* < 0.01 corrected by FDR, criterion; MNI, Montreal Neurological Institute; MFG, middle frontal gyrus; SOG, superior occipital gyrus; STG, superior temporal gyrus.

### CBF Connectivity Patterns

A total of 5 ROIs were defined as the seed regions with significant differences in the CBF between the 2 groups, including the left insula, right caudate, right SOG, left superior temporal gyrus (STG), and right middle frontal gyrus (MFG).

### Group Differences in the CBF Connectivity

**FIGURE 4:**
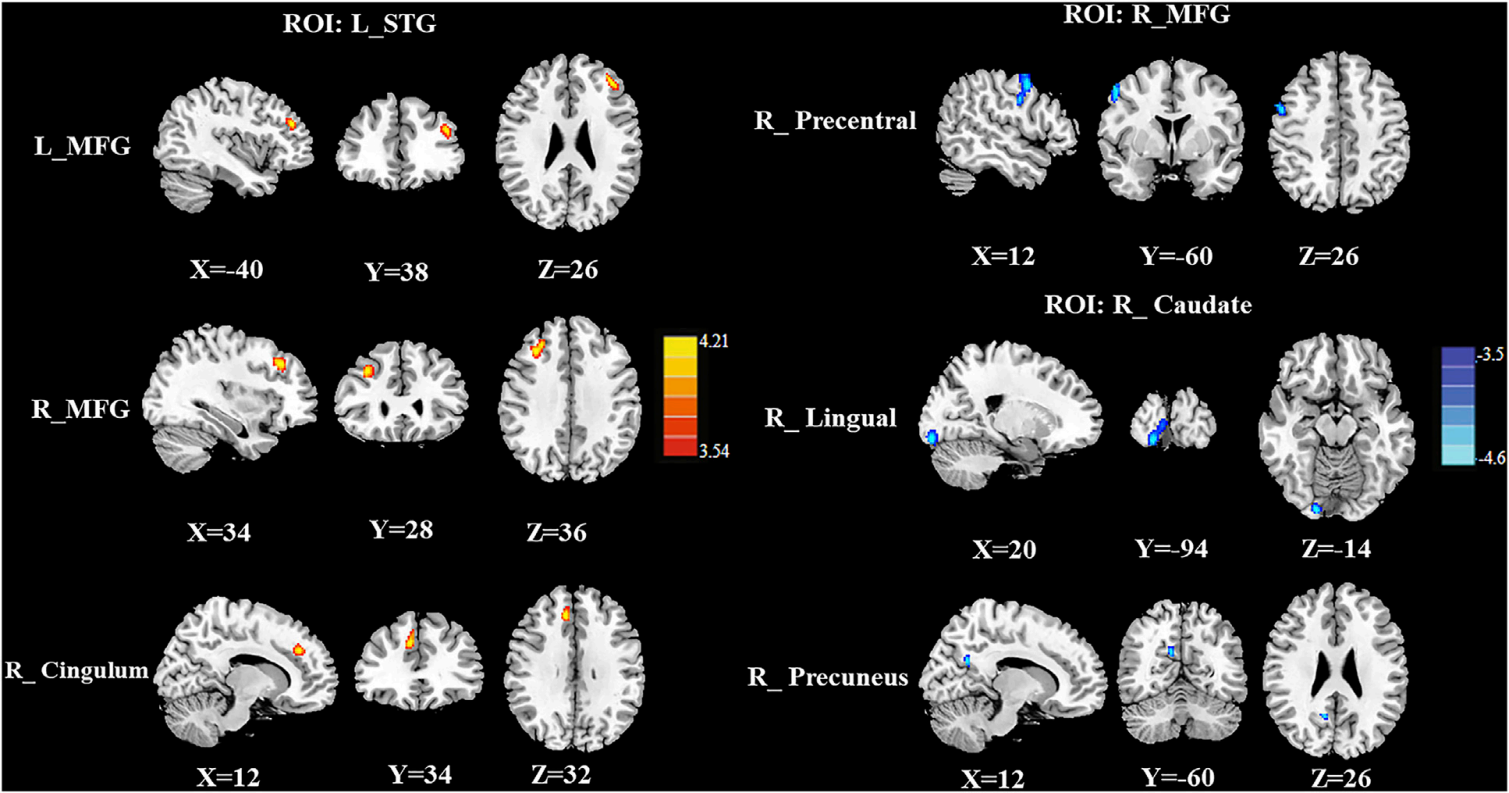
Compared with the CT (-) group, the CT (+) group exhibited decreased negative CBF connectivity between the seed ROI of the right MFG and the right precentral gyrus; these patients also showed decreased negative CBF connectivity between the seed of the right caudate and the right lingual gyrus and between the seed of the right caudate and the right preceneus. In addition, compared with CT (-) patients, CT (+) patients showed higher positive rCBF connectivity between the seed ROI of the left STG and the bilateral MFG and between the seed of the left STG and the right middle cingulum.

Group differences in the CBF connectivity are shown in Figure 4 and Table 3. Compared with the CT (-) group, the CT (+) group had decreased negative CBF connectivity between the seed ROI of the right MFG and the right precentral gyrus and decreased negative CBF connectivity between the seed of the right caudate and the right lingual gyrus and between the seed of the right caudate and the right precuneus. In addition, compared with the CT (-) group, the CT (+) group also exhibited positive CBF connectivity between the seed ROI of the left STG and the bilateral MFG and between the seed ROI of the left STG and the right middle cingulum. The seed ROI of the left insula and the right SOG in CBF did not exhibit any significant differences in the CBF connectivity between both groups.

**TABLE 3:** Brain regions with significant group differences in CBF connectivity.

ROI	Brain regions	BA	Normalized CBF		Peak mini coordinates		Peak T value	Voxels
				CT (-)	CT (+)	X, Y, Z (mm)				
L_STG	L_MFG	46	0.583 ± 0.028	0.600 ± 0.167	−40, −38, 26	4.2555	115
	R_MFG	9	0.911 ± 0.005	0.891 ± 0.298	34, 28, 36	4.2692	195
	R_Mid_Cingulum	32	0.861 ± 0.090	0.814 ± 0.086	12, 34, 32	4.3494	128
R_MFG	R_PreCG	6	0.917 ± 0.030	0.848 ± 0.055	52, 2, 46	−4.2596	219
R_Caudate	R_Precuneus	31	1.004 ± 0.150	0.922 ± 0.032	12, −60, 26	−4.0927	35
	R_LING	18	0.861 ± 0.090	1.366 ± 0.102	20, −94, −14	−4.923	177

Thresholds were set at a corrected *p* < 0.01 corrected by FDR, criterion; CBF, cerebral blood flow; ROI, region of interest; MNI, Montreal Neurological Institute; STG, superior temporal gyrus; MFG, middle frontal gyrus; PreCG, precentral gyrus; LING, lingual gyrus.

### Correlations Between Regional Normalized CBF and MoCA Scores

**FIGURE 5:**
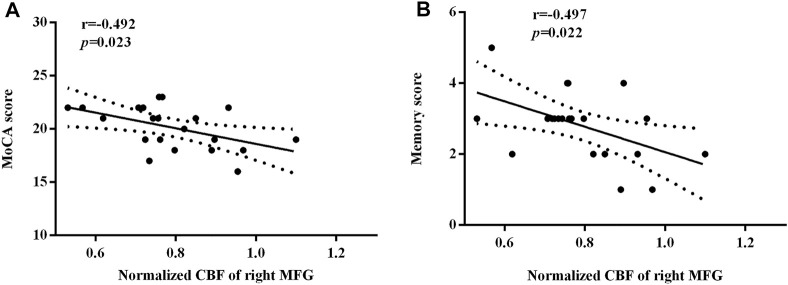
The significant correlations between the regional normalized CBF and the neurocognitive outcome. The MoCA scores **(A)** and the memory scores **(B)** were negatively correlated with the normalized CBF of the right MFG, respectively (r = −0.492, *p* = 0.023; r = −0.497, *p* = 0.022).

Figure 5 depicted significant correlations between the changes in the regional normalized CBF and MoCA scores. Only MoCA scores and memory scores were negatively correlated with the normalized CBF of the right MFG (*r* = −0.492, *p* = 0.023; *r* = −0.497, *p* = 0.022). There was no other regional CBF values correlated with the MoCA scores (include visuospatial/executive scores, naming scores, attention scores, language scores, abstraction scores, orientation scores). There were no other subcategories of the MoCA correlated with the normalized CBF of the right MFG.

## Discussion

Platinum-based chemotherapy is the first-line chemotherapy drug for pulmonary cancer and considered to induce cognitive changes. The deep-seated and complex reasons of such changes include oxidative stress, damage of DNA, hormone level changes, and immune response disorders (Ahles and Saykin, 2007). Previous studies in breast cancer have shown that docetaxel and adriamycin have peripheral anti-angiogenesis related to vasotoxicity (Vacca et al., 2002; Bar-Joseph et al., 2011). Therefore, the cerebrovascular changes caused by chemotherapy may change the cerebral blood flow. In the present study, we investigated regional CBF and CBF connectivity differences between patients who received chemotherapy and patients who did not yet receive chemotherapy, in addition to several central findings that were obtained. First, the CT (+) group had higher rCBF in the left insula, right caudate, right SOG, left STG, and right MFG, compared with the CT (-) group. Neuropsychological assessment shows that the CT (+) group performed worse than the CT (-) group, especially in the memory part. Then we identified significant aberrant CBF-connectivity within the left STG, right MFG, and right caudate. As far as we are concerned, this study is the first to utilize ASL-MRI for establishing the CBF connectivity patterns of post-chemotherapy non-small cell lung cancer patients.

MFG, STG, and insula are part of the ventral attention network which is involved in reorienting attention to salient stimuli (Suo et al., 2021). Referring to the longitudinal ASL-based CBF study in breast cancer patients, Chen et al. (2017) found significantly increased CBF in the superior frontal gyrus, inferior frontal gyrus, inferior parietal gyrus, and temporal gyrus in post-chemotherapy breast cancer patients. At the same time, their results indicated that the post-chemotherapy breast cancer patients exhibited reduced performance in the alerting and executive control attention networks which was significantly correlated with the increased CBF changes in some brain regions. These results were in partial agreement with ours. We did not identify CBF differences in the inferior frontal gyrus and inferior parietal gyrus. This heterogeneous result may result from the difference among the types of cancer, chemotherapy drugs, and the experimental methods. Ponto et al. (2015) and Nudelman et al. (2014) also found that patients with breast cancer showed high rCBF in the bilateral superior and posterior brain regions (including MFG) from the baseline to 1 month post-treatment utilized PET and ASL-MRI, respectively. Our results were in line with these studies, but more specifically, we further identified the CBF of the right MFG was negatively correlated with the MoCA scores and the memory scores. These results suggest that cognitive deficits, especially the memory function impairment, induce the compensatory hyperperfusion in the right MFG and thus contribute to the clinical symptoms. Higher CBF in the left STG in our study was inconsistent with previous findings (Chen et al., 2017; Feng et al., 2020b).

In addition, the task fMRI study on childhood acute lymphoblastic leukemia indicated that increased brain activation (attention network task alerting) related to higher methotrexate exposure in the frontal area, insula, caudate, and anterior cingulate (Fellah et al., 2019). Insula is thought to be related to emotions and consciousness. A previous study reported that patients who accept chemotherapy showed persistent symptoms of anxiety and depression over time, and changes in psychological symptom scores were significantly positively correlated with changes in FC between the left hippocampus and left insula (Feng et al., 2020a; Hu et al., 2020). Omran et al. (2021) found the brain plays a prominent role in the chemotherapy-induced peripheral neuropathy. The likely reason is brain hyperactivity and neuroinflammation may cause large-scale brain networks in several brain regions including insula. Therefore, the insula may be responsible for changes in brain functional connectivity, CBF, and peripheral neuropathy caused by chemotherapy. Therefore, we believe that the insula is a sensitive region to explore the effects of chemotherapy on the brain.

Caudate is a part of the cortico-striato-thalamo-cortical loop, which is involved in the hyperkinetic and hypokinetic movement disorders and mental disorders (ırak et al., 2020). Chen et al. (2020) identified significant decreasing mean fractional amplitude of low-frequency fluctuation trend in the left caudate in the CT (-) group compared with the CT (+) group in the population with breast cancer. We suspected that caudate may play a role in the cognitive impairment of cancer patients, which may provide evidence in the change in CBF of the caudate nucleus in patients with lung cancer after chemotherapy. With the use of PET, Fang et al. (2016) demonstrated that patients with cancer had abnormal CBF in their SOG, and altered CBF was linked to cognitive function. Different from these results, we did not identify any significant correlation between the CBF values in SOG and the cognitive scores. This inconsistent result may be due to the difference between the subject and the experimental method.

CBF connectivity is a meaningful method to evaluate the functional networks within brain regions (Li et al., 2020). The CBF connectivity can be obtained by calculating the CBF correlation coefficient between a group of brain regions. It is different from BOLD connectivity, which was calculated by measuring the time correlation between BOLD signal fluctuations across brain regions. CBF connectivity mainly manifested the difference among groups. It has a physiological implication of temporally coordinated metabolism and has been applied to investigate Parkinson’s disease and migraine (Zhang D. et al., 2020; Shang et al., 2021). To our knowledge, no research has yet investigated the CBF connections of CRCI. In the present study, we identified CBF disconnections between the right MFG and the right precentral gyrus. This result was consistent with previous BOLD connectivity study in breast cancer (Tao et al., 2017). Precentral gyrus is a part of the premotor area and the supplementary motor area, which integrate the incoming sensory information from the outside world and the current state of bod (Hooks, 2017). Existing studies have suggested that changes in the neurovascular coupling and cerebral perfusion patterns in the motor region are related to motor development (Song et al., 2020). We also observed decreased negative CBF connectivity between the right caudate and the right lingual gyrus as well as the right preceneus. The lingual gyrus is part of the primary visual cortex and the precuneus is a part of the DMN. Aberrant functional connectivity in these two regions has been reported in previous studies of breast cancer (Apple et al., 2018). The disconnection between the right MFG as well as the right caudate and these regions may be linked with the functional deficits in the cognitive integration in the CT (+) group.

In addition, we found a higher CBF connectivity between the left STG and bilateral MFG as well as the right cingulum in the CT (+) patients. STG and MFG belong to the ventral attention network, higher rCBF and increased CBF connectivity were found simultaneously in both regions in our study. We speculate that the ventral attention network may be a sensitive network affected by chemotherapeutic drugs. The cingulum is a tract of association fibers lying within the cingulate gyrus and connects the callosal and hippocampal convolutions of the brain, and damage of this tract can cause abnormal emotional and cognitive behaviors. Thus, the increased connections between the seed ROI of the left STG and these regions are more likely to be suggested as compensatory for dysfunctions. These results indicated that CBF connectivity alterations may be a compensation for realizing certain network functions in patients with non-small cell lung cancer after chemotherapy, which provided a new research perspective for CRCI.

Several limitations should be acknowledged. First, we recruited a relatively small sample, which may affect the statistical reliability of our results. In further research, we hope to expand the sample size and obtain more reliable results. In addition, we will further refine intergroup confounding factors in further experiments. Second, a healthy control group in our research was not included. The reason is our current research mainly focused on the difference between the CBF and CBF connection between post-chemotherapy and pre-chemotherapy patients with non-small cell lung cancer. In future research, we will include a healthy control group to further explore whether lung cancer itself has an impact on CBF changes. Third, our study is a relatively small sample and cross-sectional experimental design. Thus, it is difficult to make direct causal inferences about the relationship between CBF changes and cognitive impairment. Thus, further longitudinal studies involving a larger number of participants are needed to confirm the present conclusions. At last, CBF connectivity is calculated by analyzing the inter-regional CBF correlation between the test groups and could not be used for additional correlation analysis with cognitive or clinical measurements. This problem will be settled with the development of multi-latency ASL techniques and advanced analysis.

In conclusion, our study used ASL-MRI to detect CBF and CBF connectivity differences between post-chemotherapy non-small cell lung cancer patients and pre-chemotherapy patients. These findings highlighted the necessity of studying the underlying neuropathology of cognitive impairment in lung cancer patients from the perspective of the regional and inter-regional characteristics of CBF in the resting status. Nonetheless, it is an exploratory work and more tests are required to verify whether it is suitable for replication.

## Data Availability

The original contributions presented in the study are included in the article/Supplementary Material, further inquiries can be directed to the corresponding authors.
